# Research on the Method of Predicting Corrosion width of Cables Based on the Spontaneous Magnetic Flux Leakage

**DOI:** 10.3390/ma12132154

**Published:** 2019-07-04

**Authors:** Yinghao Qu, Hong Zhang, Ruiqiang Zhao, Leng Liao, Yi Zhou

**Affiliations:** 1 College of Civil Engineering, Chongqing Jiaotong University, Chongqing 400074, China; 2 College of Materials Science and Engineering, Chongqing Jiaotong University, Chongqing 400074, China; 3 Chongqing Yapai Bridge Engineering Quality Inspection Co., Ltd., Chongqing 401120, China

**Keywords:** spontaneous magnetic flux leakage, corrosion width, cable corrosion detection, *D*_x_–*y* curves

## Abstract

The detection of cable corrosion is of great significance to the evaluation of cable safety performance. Based on the principle of spontaneous magnetic flux leakage (SMFL), a new method for predicting the corrosion width of cables is proposed. In this paper, in order to quantify the width of corrosion, the parameter about intersecting point distance between curves of magnetic flux component of *x* direction at different lift off heights (*D*_x_) is proposed by establishing the theoretical model of the magnetic dipole of the rectangular corrosion defect. The MATLAB software was used to analyze the influencing factors of *D*_x_. The results indicate that there exists an obvious linear relationship between the *D*_x_ and the *y* (lift off height), and the *D*_x_–*y* curves converge to near the true corrosion width when *y* = 0. The 1/4 and 3/4 quantiles of the *D*_x_–*y* image were used for linear fitting, which the intercept of the fitting equation was used to represent the predicted corrosion width. After the experimental study on the corrosion width detection for the parallel steel wire and steel strand, it is found that this method can effectively improve the detection accuracy, which plays an important role in cable safety assessment.

## 1. Introduction

As the main stressed components of cable-stayed bridges and suspension bridges, cables play an important role in the whole bridge system [1]. After the cable sheath is damaged, the cable will corrode under long-term action, which will affect the bearing capacity of the cable and the overall safety of the bridge. Therefore, the corrosion detection of the cable is very important [2].

At present, corrosion detection methods are divided into damage methods, semi-damage methods and non-destructive methods [3]. As the important structure of the bridge, the damage of protective layer will affect its bearing capacity and durability, so non-destructive methods should be given priority. The common methods of non-destructive corrosion detection include X-ray detection [4], acoustic emission detection (AE) [5,6,7], magnetic particle detection [8], eddy current detection [9,10,11], magnetic flux leakage detection (MFL) [12,13,14], etc. All of these methods can be effective to some extent in the detection of rust, but they all have shortcomings. X-ray detection is inefficient, expensive and easy to cause radiation pollution. The magnetic particle detection and eddy current testing can only be used to detect defects on or near the surface of ferromagnetic materials. The accuracy of AE detection for defect size boundary is not enough. The MFL detection requires saturation magnetization of components, which makes the equipment more complex, and the detection speed is slower. The corrosion of the cable occurs on the internal parallel wire strands or steel strands. With the corrosion defects hidden under the thick PE sheath, it is difficult to make the accurate evaluation by conventional methods.

The principle of spontaneous magnetic flux leakage (SMFL) can be mainly expressed that ferromagnetic materials will be magnetized in the geomagnetic field for a long time. Corrosion of ferromagnetic materials will result in discontinuity of the surface of the material, which also leads to the mutation of magnetic permeability and the distortion of magnetic field lines. Macroscopically, it is reflected as the magnetic leakage phenomenon at the defect. The law shows that the magnetic induction intensity component of SMFL along the length direction of the component appears the extreme value, while the component in the direction of lift off height crosses the zero point [15]. Compared with traditional non-destructive testing methods, the SMFL detection has the following advantages [16,17]:

- Because SMFL detection does not rely on external magnetic field excitation, only a high-precision magnetic field probe can ensure faster detection speed.
- The SMFL field can penetrate the protective layer, so the detection signals can be obtained outside the protective layer. It is a non-destructive method that can detect hidden defects.
- The SMFL magnetic signal has a specific response to the size of the corrosion defect, and the parameters of corrosion defects can be quantified by it.

In two-dimensional space, the defects caused by corrosion can be described by corrosion depth and corrosion width, which are important indicators affecting the bearing capacity of the structure. In view of the problem of size detection of corrosion defects, many experts have conducted a certain degree of research. Xia [17] studied the correlation between the corrosion depth of cables and the peak signal of SMFL field. Grinzato [18] used infrared thermal imaging technology on the shapes of the defect detection of the actual boiler. Zhang [16] analyzed the correlation between the SMFL and corrosion characteristics. Zhang [19] quantitatively analyzed the relationship between crosssection loss of cable and magnetic signal by establishing a finite element model. Bacchelli [20] studied the reconstruction of the damaged profile of a thin metallic plate, which is possible by measuring an electrostatic current properly induced by a potential in an accessible part of the boundary. Han [21] estimated the location, shape and size of the corrosion area by analyzing the surface temperature distribution of the sample. Caleyo [22] proposed a new Bayesian method to analyze the external corrosion data of underground non-gas pipelines, which allows the statistical distribution of the density and size of external corrosion defects to be estimated based on the corrosion data samples collected from the excavation site along the pipeline being inspected. Satyarnarayan [23] studied the nondispersive propagation of ultrasonic guided wave high-order mode cluster (HOMC) along the circumference of a hollow cylinder and its interaction with defects of different sizes in the supporting area of the pipe. Snarskii [24] uses a three-dimensional integral equation to describe the MFL field, which can be used to calculate the MFL field for a given defect shape. Liu [25] studied the circumferential excitation method by using the linear magnetic dipole model, and established the detection model of the axial crack in the pipeline, which the non-uniform magnetic leakage field generated by the circumferential surface excitation can effectively detect the depth and width characteristics of the narrow axial crack in the pipeline.

In this paper, combined with the magnetic dipole model and numerical analysis, the principle of SMFL was used to put forward a parameter about intersecting point distance between curves of magnetic flux component of *x* direction at different lift off heights (*D*_x_) and the sensitivity analysis was carried out. According to the analysis results of *D*_x_–*y* curves, a method for detecting the corrosion area was proposed. At last, the corrosion detection test was carried out on the parallel wire strands and steel strands, where the test results were verified, and errors were analyzed by using this method.

## 2. Materials and Methods 

### 2.1. Theoretical Model

In the rectangular corrosion defect model, the corrosion width and depth are 2*b* and *h*, and the lift off height is *y*. Under the action of geomagnetic field, the magnetization of ferromagnetic materials after they are magnetized is *M*, which can be expressed as a function of *x* and *y*. The magnetic charge density (*ρ*) represents the density of magnetization intensity at any point inside the material, so it can be expressed as:

(1) $$\rho =-\nabla M=-(\frac{\partial M_{x}}{\partial x}+\frac{\partial M_{y}}{\partial y}).$$

**Figure 1:**
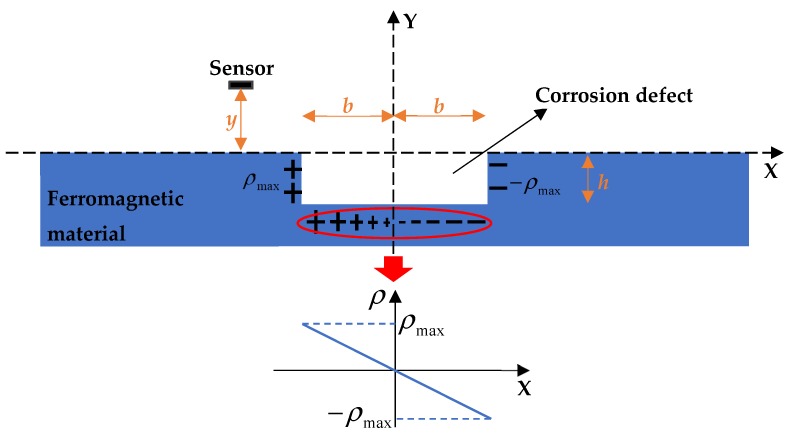
The calculated diagram of magnetic dipole model with the rectangular defect. *X*: the length direction of ferromagnetic material; *b*: the half of corrosion width; *h*: the corrosion depth; *ρ*_max_: the magnitude of magnetic charges on both sides of the defect, and the plus and minus symbols represent the plus and minus of magnetic charges; the distribution of magnetic charges at the bottom of the defect is shown in the red circle, which is linearly distributed along the *x* direction; *y*: the lift off height.

For the slender bar, the direction of magnetization is mainly along the length direction, while the other directions can be considered as a constant, because of its small size. Therefore, it can be considered that the *ρ* of the same size and opposite polarities are produced on the wall of the defect, but its linear at the bottom [15], as shown in Figure 1. The *ρ* can be expressed as a piecewise function as follows:

(2) $$\rho =\{\begin{array}{cc} \rho_{\max} & x=-b\\ \frac{\rho_{\max}}{b}x & -b<x<b\\ -\rho_{\max} & x=b \end{array}.$$

Based on the vector superposition and integral operation, we can get the magnetic field strength along the length of the bar (*H*_x_) as follows:

(3) $$H_{x}=\int_{-h}^{0}dH_{x=-b}+\int_{-h}^{0}dH_{x=b}+\int_{-b}^{b}dH_{-b<x<b},$$

(4) $$\begin{array}{l} H_{x}=\frac{\rho_{\max}}{2\pi \mu_{0}}[\arctan\frac{y+h}{x+b}-\arctan\frac{y}{x+b}+\arctan\frac{y}{x-b}-\arctan\frac{y+h}{x-b}+2\\ -\frac{y+h}{b}(\arctan\frac{b-x}{y+h}+\arctan\frac{b+x}{y+h})+\frac{x}{2b}\ln\frac{{\left(b-x\right)}^{2}+{\left(y+h\right)}^{2}}{{\left(b+x\right)}^{2}+{\left(y+h\right)}^{2}}] \end{array}$$

**Figure 2:**
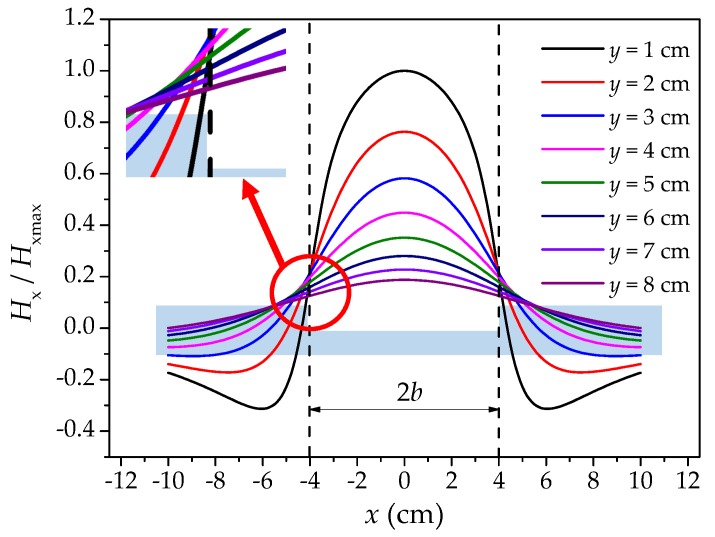
The normalized magnetic field intensity signal of the theoretical model of the rectangular defect. *X*: the coordinate of length direction of the ferromagnetic material with the center of corrosion defect as the origin; *H*_x_/*H*_xmax_: the ratio of the magnetic field intensity to its maximum value; *b*: the half of corrosion width of the defect; *y*: the lift off height.

In Equation (4), the magnetic charge density *ρ*_max_ is the product term. Because *ρ*_max_ is not a function of *x* and *y*, it can only enlarge and reduce *H*_x_ and cannot change the distribution law of *H*_x_. However, the terms within square brackets directly determine the shape of *H*_x_ curve. In order to obtain the SMFL field distribution of rectangular defects, the defect width *b*, the depth *h* and $\rho_{\max}/\mu_{0}$ were assumed to be 4 cm, 2 cm and 1. Along the *x*-axis, from −10.0 to 10.0 cm, and the lift off value *y* from 1 cm to 8 cm, the *H*_x_ was calculated, as shown in Figure 2, after normalization. The parameters of the calculation are shown in Table A2. 

As for Figure 2, two intersections can be formed for each of the two curves with different *y*, and the two intersections are symmetrical to the center of the corrosion area. Through magnification observation, these intersection points are distributed discreetly at the boundary of the corrosion area, and the location of the intersection points deviates from the corrosion boundary with the increase of *y*. Therefore, the relationship between the distribution of these intersections and the boundary of the corrosion region is analyzed. The existence condition of the intersection point is shown below.

(5) $$H_{x\left(y=y_{1}\right)}=H_{x\left(y=y_{2}\right)},$$

where the *y*_1_ and the *y*_2_ are two different lift off heights.

### 2.2. Model Analysis

**Figure 3:**
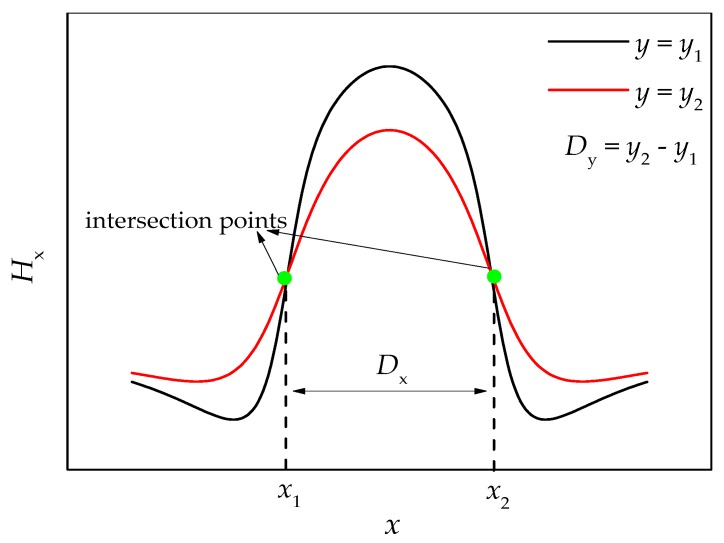
The schematic diagram of what *D*_x_ means. *H*_x_: magnetic field intensity component; *x*_1_, *x*_2_: the *x* coordinates when two *H*_x_ curves intersect. *D*_x_: the distance between two intersection points; *D*_y_: the difference between two lift off heights.

In order to describe more directly the relationship between the intersection point and the corrosion boundary, a parameter $D_{x}=\left|x_{1}-x_{2}\right|$ is introduced here, which is defined as the distance between the two intersection points generated by the *H*_x_ curves at different *y*, as shown in Figure 3.

**Figure 4:**
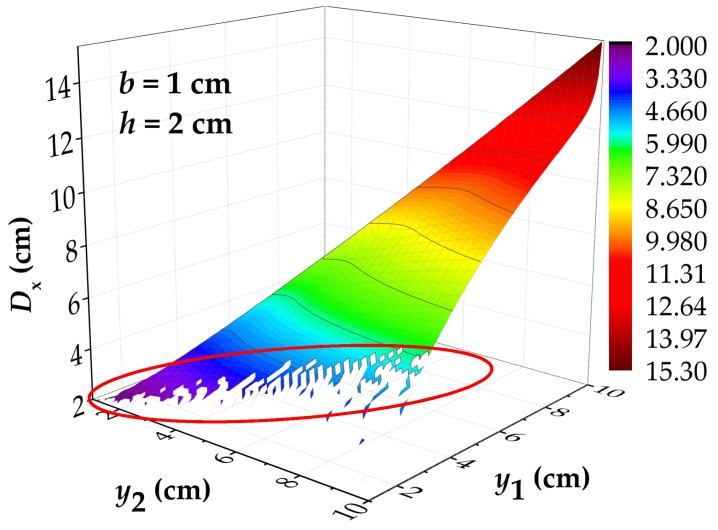
When *b* = 1 cm and *h* = 2 cm, the calculated results of *D*_x_ under different values of *y*_1_ and *y*_2_. The value of *D*_x_ tends to increase with the increase of *y*_1_ and *y*_2_. A blank area (circled by the red line) means that *D*_x_ has no solution. *D*_x_: the intersecting point distance between curves of magnetic flux component of *x* direction at different lift off heights; *y*_1_ and *y*_2_: the lift off height; *b*: the half of corrosion width.

Since *D*_x_ comes from Equation (5), it is a function of the four parameters of *b*, *h*, *y*_1_ and *y*_2_, which is expressed as *D*_x_ = f (*b*, *h*, *y*_1_, *y*_2_). Equation (5) is solved numerically with MATLAB software. The value of *x* is recorded when the convergence condition is satisfied. The convergence condition of the numerical calculation is:

(6) $$f=\left|H_{x\left(y=y_{1}\right)}-H_{x\left(y=y_{2}\right)}\right|<\left|H_{x\left(y=y_{i}\right)}\right|\times e\;i=1,2,$$

where the *e* is the accuracy of convergence, which the value is set as 0.01. On the premise of ensuring *y*_1_ > *y*_2_, the range of values of *y*_1_ and *y*_2_ and the step length of value are set as 0 cm to 10 cm and 0.1 cm, which the other parameters *b* and *h* are set as 1 cm and 2 cm. After calculation, a 3D figure of *D*_x_–*y*_1_–*y*_2_ is drawn, as shown in Figure 4. The parameters of the calculation are shown in Table A3.

As shown in Figure 4, the value of *D*_x_ tends to increase with the increase of *y*_1_ and *y*_2_. Then, the value of *D*_x_ at this time is exactly equal to 2 cm (the corrosion width) when the *y*_1_ and *y*_2_ approach to 0 cm at the same time. With the *y*_1_ remaining unchanged and *y*_2_ increasing gradually, the *D*_x_ decreases to a certain extent. However, a blank area (circled by the red line) will appear in the figure, which means that *D*_x_ has no solution at this time. The reason for this phenomenon is that the value of convergence precision is too small so as to cause that the value of *x* cannot satisfy the Equation (6). From the above analysis, it can be found that the value of *D*_x_ is affected by the difference between *y*_1_ and *y*_2_. Therefore, *D*_y_ is defined as *y*_2_ − *y*_1_, and *y*_1_ is redefined as *y*, then *D*_x_ = f (*b*, *h*, *y*, *D*_y_). In order to further obtain the distribution law of *D*_x_, the four parameters were separately studied here.

#### 2.2.1. Influence of Parameters *b* and *h* on *D*_x_

**Figure 5:**
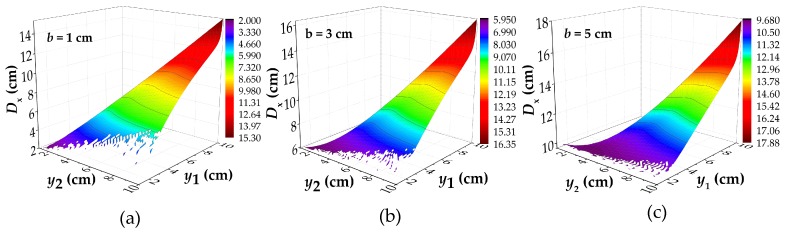
The calculated results of *D*_x_ under the different values of *b*: (**a**) the calculated results of *D*_x_ under *b* = 1 cm; (**b**) the calculated results of *D*_x_ under *b* = 3 cm; (**c**) the calculated results of *D*_x_ under *b* = 5 cm; *D*_x_: the intersecting point distance between curves of magnetic flux component of *x* direction at different lift off heights; *y*_1_ and *y*_2_: the lift off height; *b*: the half of corrosion width.

**Figure 6:**
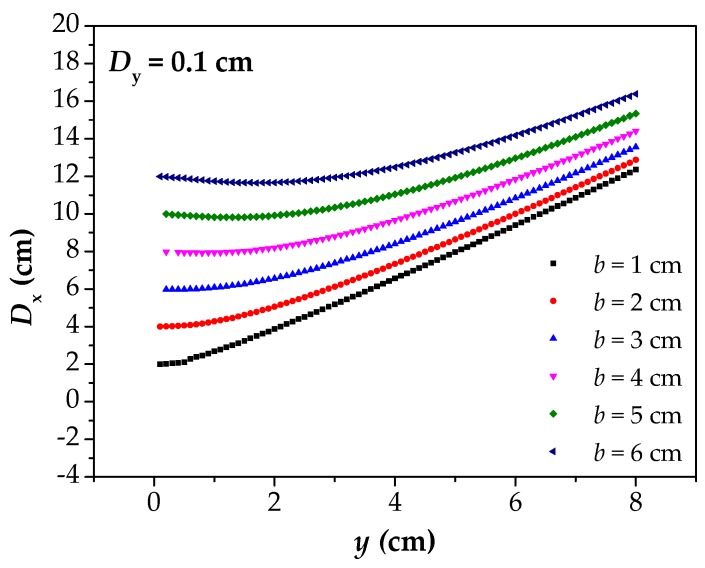
When *D*_y_ is 0.1, the calculated results of *D*_x_ under different *b*. *D*_x_: the intersecting point distance between curves of magnetic flux component of *x* direction at different lift off heights; *D*_y_: the difference between two lift off heights; *b*: the half of corrosion width.

The values of *b* and *h* are set as 1 cm to 6 cm and 2 cm, and the partial results are shown in Figure 5. All of the parameters of calculation are shown in Table A4. For different values of *b*, the distribution of *D*_x_–*y*_1_–*y*_2_ images are basically the same. With the increase of the *b*, the number of unsolved points in the image decreases obviously. The reason for this phenomenon is that the $\left|H_{x\left(y=y_{i}\right)}\right|$ in Equation (6) increases with the *b* increasing, which indirectly reduce the convergence condition. In order to ensure that the data has a solution, the value of *D*_y_ set as 0.1 cm for analysis, as shown in Figure 6. 

The image of the Figure 6 reflects the relationship between *D*_x_ and *y* under different corrosion widths. The *D*_x_–*y* curves reflect the fact that the *D*_x_ finally converges to 2*b* in all cases with the *y* approaching to 0. Moreover, the *D*_x_–*y* curves gradually flatten with the decrease of the *y*, and this phenomenon becomes more and more obvious with the increase of the *b*. At the same time, the *D*_x_–*y* curves increase linearly with the increase of the *y*, which remain parallel.

**Figure 7:**
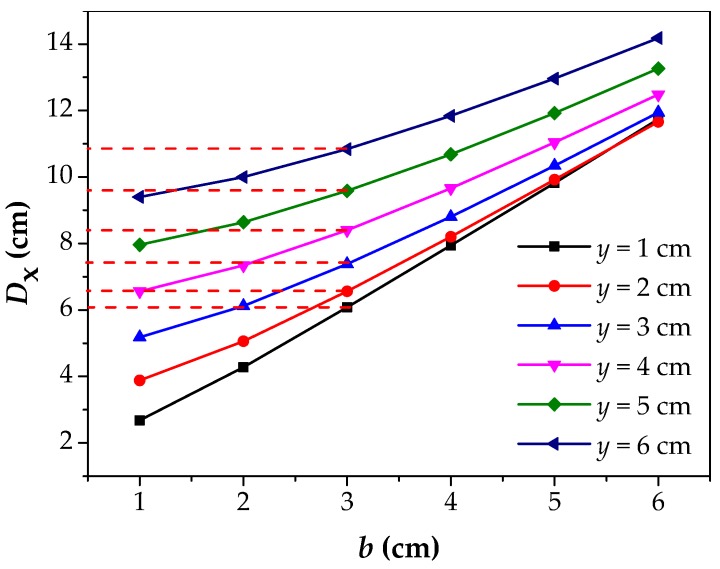
The relationship between *D*_x_ and *b* under different *y*. *D*_x_: The intersecting point distance between curves of magnetic flux component of *x* direction at different lift off heights; *b*: the half of corrosion width; *Y*: the lift off height.

Further, the *D*_x_–*b* curves are drawn when the value of *y* is set as 1 cm to 6 cm, as shown in Figure 7. This image reflects the change of the *D*_x_ with respect to the *b*. The curves show a same rule that the *D*_x_ increases with the increase of the *b*, which show a linear relationship. At the same time, the growth rate, namely the slope of the curves, decreases slowly with the increase of the *y*, but the overall situation is basically similar. The above phenomenon indicates that the parameter *D*_x_ has a good positive linear correlation with the *b*. Moreover, the figure also reflects the error that the *D*_x_ is used to estimate 2*b* (the corrosion width). Particularly, the analysis is carried out when the value of the *b* is 3 cm. Ideally, the error is zero when *D*_x_ is equal to 2*b* = 6 cm. It can be seen from the figure that the larger the *y* is, the farther the value of *D*_x_ (marked by the red dotted line) deviates from the true value, and the larger the error will be. The slope of the curve is close to 2 with the value of the *y* set as 1 cm, that is, *D*_x_ is approximately 2*b*, which indicates that the smaller the *y* is, the more favorable it is for *D*_x_ to speculate the corrosion width.

**Figure 8:**
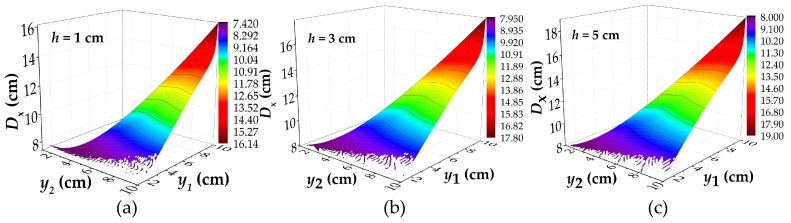
The calculated results of *D*_x_ under the different values of *h*: (**a**) the calculated results of *D*_x_ under *h* = 1 cm; (**b**) the calculated results of *D*_x_ under *h* = 3 cm; (**c**) the calculated results of *D*_x_ under *h* = 5 cm; *D*_x_: the intersecting point distance between curves of magnetic flux component of *x* direction at different lift off heights; *y*_1_ and *y*_2_: the lift off height; *h*: the depth of corrosion defect.

**Figure 9:**
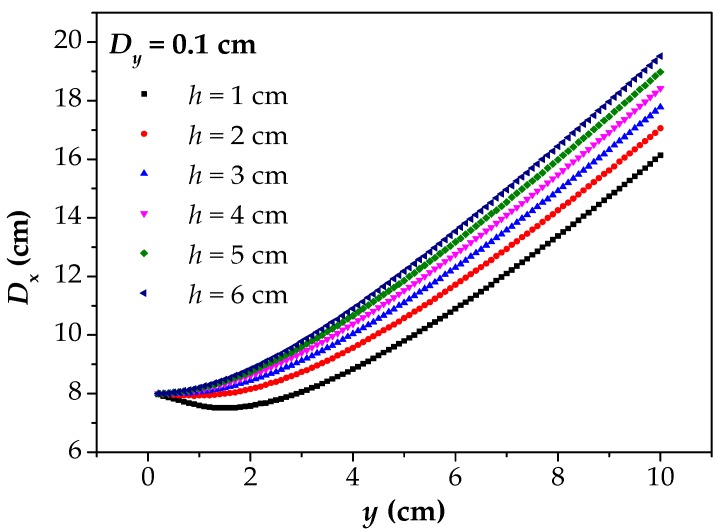
When *D*_y_ is 0.1, the calculated results of *D*_x_ under different *h*. *D*_x_: the intersecting point distance between curves of magnetic flux component of *x* direction at different lift off heights; *h*: the depth of corrosion defect; *D*_y_: the difference between two lift off heights; *y*: the lift off height.

The relationship between *D*_x_ and *h* were analyzed for *b* = 4 cm, and *h* = 1 cm to 6 cm, and partial results are shown in Figure 8. All of the parameters of calculation are shown in Table A5. Figure 8 reflects the relationship between *D*_x_ with different lift off heights. It can be seen from the image that for the different *h*, the overall trend of *D*_x_ is basically the same. As the same with the Figure 5, the whole curves show a same trend of divergence with the increase of *y*_1_ and *y*_2_, but they all converge to the same value with the decrease of *y*_1_ and *y*_2_. In the same processing method, the results of two-dimensional data are shown in Figure 9.

**Figure 10:**
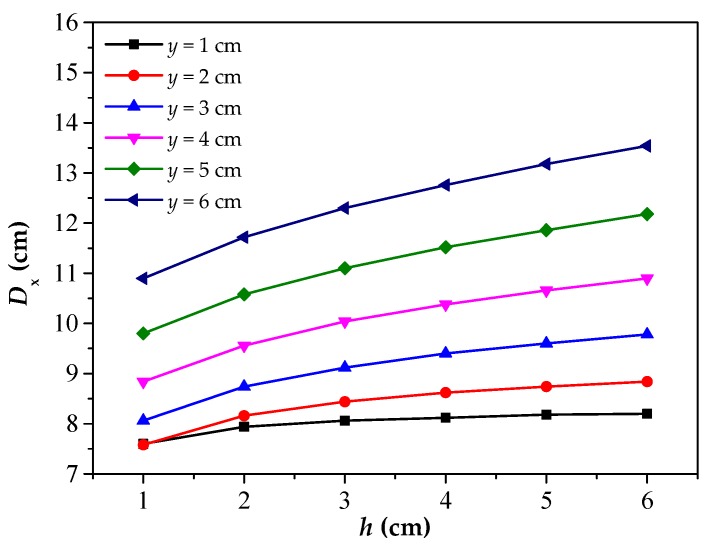
The relationship between *D*_x_ and *h* under different *y*. *D*_x_: the intersecting point distance between curves of magnetic flux component of *x* direction at different lift off heights; *h*: the depth of corrosion defect; *Y*: the lift off height.

The image of Figure 9 reflects the relationship between *D*_x_ and *y* under different *h*. In Figure 9, *D*_x_ converges to the 2*b* with the *y* decreasing. However, for different *h*, the trend of convergence is not consistent. With the *h* decreasing, the *D*_x_–*y* curves present a trend of decreasing first, then increasing, and finally converge to the true value. However, with the *h* increasing, the *D*_x_–*y* curves are close to a straight line. In order to more intuitively obtain the relationship between *D*_x_ and *h*, the data in Figure 9 is extracted here to obtain the *D*_x_–*h* curves, as shown in Figure 10.

Figure 10 reflects the change rule of *D*_x_ with the *h*. In Figure 10, the *D*_x_ increases slowly with the increase of *h*, basically close to a trend of linear increase. Moreover, the value of *D*_x_ increase more quickly with the *y* increasing, which can be reflected from the curves for the change of the slope. The slope close to 0 when *y* = 1 cm, and the corrosion depth has less effect on the *D*_x_ and more advantage to detection when *y* is little.

#### 2.2.2. Influence of Parameters *y* and *D*_y_ on *D*_x_

**Figure 11:**
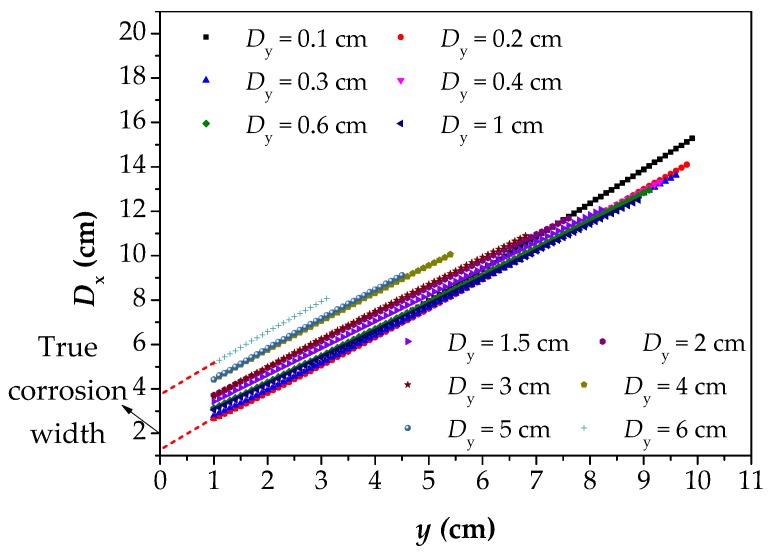
The calculated results of *D*_x_ with different values of *D*_y_. *D*_x_: the intersecting point distance between curves of magnetic flux component of *x* direction at different lift off heights; *D*_y_: the difference between two lift off heights; *y*: the lift off height.

The *y* and *D*_y_ are parameters for the lift off heights and subjective factors affecting *D*_x_, which can be controlled artificially in the detection process. However, in practice, there exist two reasons to limit the lift off height. The one is that a certain distance between the cable body and the PE jacket surface, and the other one is the limit due to the detector’s own structure such as sensor shell and so on. In order to further obtain an obvious rule, the corrosion parameters *b* = 1 cm and *h* = 3 cm are taken as constants to study, and the *D*_x_–*y* curves under different *D*_y_ working conditions are drawn, as shown in Figure 11.

Figure 11 reflects the change rule of *D*_x_ with *y* from 1 cm to 10 cm. In Figure 11, *D*_x_ increases linearly with the increase of the *y*. It further indicates that the deviation of the *D*_x_ from the correct corrosion width will increase with the increase of the *y*, and the deviation degree is distributed linearly. For different values of *D*_y_, the slopes of the curves are basically the same, which indicates that the influence of the *y* on *D*_x_ is basically the same. In other words, the influence of *y* on *D*_x_ is stable. Moreover, with the *D*_y_ increasing, the number of data points decreases, because of the definition of the *D*_y_. When *y* = 1, it can be found that the *D*_x_–*y* curves tend to be close to the true corrosion width. However, with the decreasing of the *y* to 0 (the dotted red line), the upper limit of *D*_x_ is greater than the true corrosion width, while the lower limit is less than it. 

**Figure 12:**
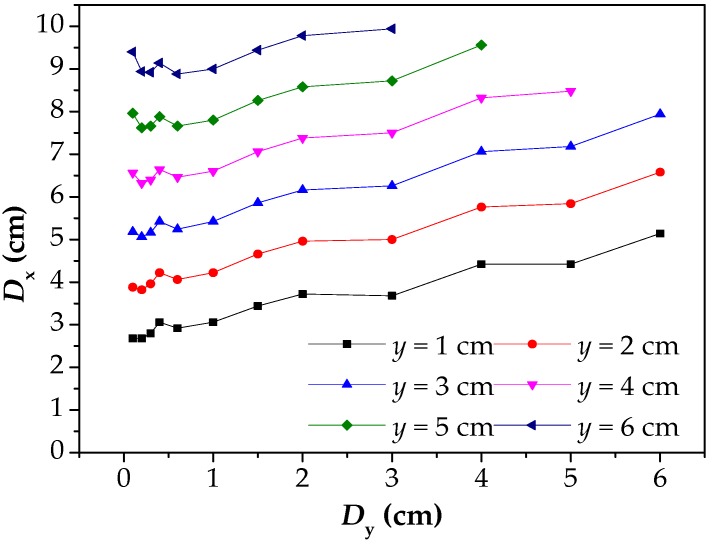
The relationship between *D*_x_ and *D*_y_ under different *y*. *D*_x_: the intersecting point distance between curves of magnetic flux component of *x* direction at different lift off heights; *D*_y_: the difference between two lift off heights; *y*: the lift off height.

In Figure 11, the *D*_y_ has no effect on the slope of *D*_x_–*y* curves, but it will affect the position of the curves. Therefore, in order to illustrate the influence of *D*_y_ on *D*_x_, the data in Figure 11 are also processed to obtain the *D*_x_–*D*_y_ curves, as shown in Figure 12. In this figure, the *D*_x_ increases linearly with the increase of the *D*_y_, but the slopes of curves are little, which indicates that the *D*_y_ has little influence on *D*_x_. At the same time, for different elevations of *y*, the *D*_x_–*D*_y_ curves are essentially the same, and it indicates that the laws of *D*_x_ and *D*_y_ are also stable.

### 2.3. Detection Method

**Figure 13:**
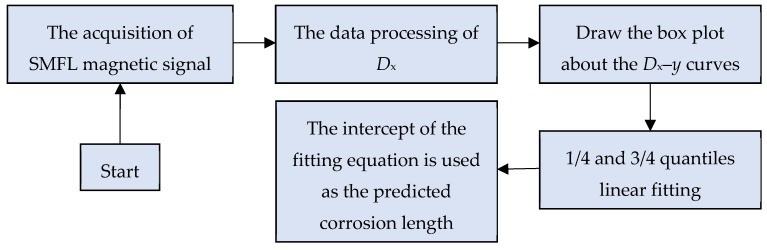
The flow chart of corrosion width prediction method.

Based on the analysis of the influence of four parameters of *b*, *h*, *y* and *D*_y_ on *D*_x_, it is found that *D*_x_ maintains a high linear relationship with *y* under the same *b* and *h*. Therefore, in theory, the value of the *D*_x_ when *y* = 0 can be predicted by linear fitting as the predicted value of the corrosion width. Because the *b* and *h* are parameters that we need to test and cannot be determined in advance, they are not taken into consideration when the method is proposed. However, through the previous analysis, the *D*_x_–*y* curves present a similar rule in the different *b* and *h*, so the method of predicting the corrosion width through the *D*_x_–*y* curves is also applicable to each corrosion condition. The method flow is shown in Figure 13.

**Figure 14:**
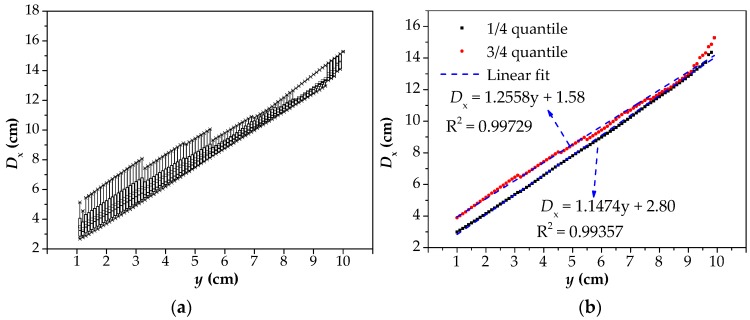
The data processing method: (**a**) the box diagram obtained after data processing; (**b**) Linear fitting of 1/4 and 3/4 quantiles. *D*_x_: the intersecting point distance between curves of magnetic flux component of *x* direction at different lift off heights; *y*: the lift off height; R^2^: goodness of fit.

Before linear fitting, all data in Figure 11 should be processed and drawn into the box plot, as shown in Figure 14a. The values of the 1/4 and 3/4 quantiles of each box should be taken as the final fitting data to obtain a range of predicted values. The reason for adopting this method is that the *D*_x_–*y* curves do not maintain linear rules, but gentle trends when the value of *y* is small, as shown in Figure 6 and Figure 9. If only the *D*_x-y_ curves with the smallest *D*_y_ is taken as fitting data, the predicted values are often smaller than the actual corrosion width. Therefore, the data under multiple values of *D*_y_ are integrated together to draw a box diagram, and the influence factors of *D*_y_ on *D*_x_ are also taken into account. Moreover, 1/4 and 3/4 quantiles were adopted to eliminate the influence of overlarge or too small data on the results, further narrowing the prediction range. The optimal R^2^ was adopted as the fitting standard. In Figure 14b, the R^2^ was approximately close to 1, which indicates that the fitting effect was the best.

The predicted results and errors are shown in Table 1. Finally, the value interval of *D*_x_ is the value of the linear fitting curve of 1/4 and 3/4 quantiles when *y* = 0. Under the corrosion conditions of this model (2*b* = 2 cm, *h* = 1 cm), the value range of *D*_x_ is 1.58 cm to 2.80 cm, and the maximum deviation is 40%. Compared with the *D*_x_ = 5.18 cm when *y* = 3 cm and *D*_y_ = 0.1 cm, the accuracy is improved by at least 119%. Because the distance between the ferromagnetic material inside the cable and the sensor chip, including the thickness of PE sheath and sensor shell is generally about 3 cm, the values of *D*_x_ when *y* = 3 cm are adopted for comparison. Hence, the result will greatly deviate from the true corrosion width if it is directly used as the detection result without adopting this method to predict the corrosion width.

Through the analysis of the model, the relationship between *D*_x_ and each parameter is obtained, and a method to improve the precision is summarized. The greatest value of this method is that it can predict the range of corrosion length and improve the detection accuracy when the minimum lifting height is limited, especially for the hidden engineering such as cable member with PE sheath. Therefore, the corrosion detection experiment of unit members of cables (steel strand and parallel wire strand) as samples was carried out to verify the feasibility of this method.

## 3. Results and Discussion

### 3.1. Experiment

Parallel wire strands and steel strands were used as specimens. The parallel wire strands were made of 7 and 19 galvanized steel wires with a length of 1.5 m, denoted as P1 and P2, respectively. The steel wire strands were corroded locally, that is, the corrosion only starts from one side of the steel wire strand section, which the corrosion width was controlled to be 4 cm. The steel strand was denoted as S1 with the length of 1 m, and the overall uniform corrosion is adopted, that is, the steel strand was corroded from the outside to the inside along the entire section, which the corrosion width is controlled as 8 cm. The parameters of the experimental specimens are shown in Table 2.

The experiment was divided into two steps. The first step was the corrosion of specimens, and the second step was the collection of the SMFL signal.

**Figure 15:**
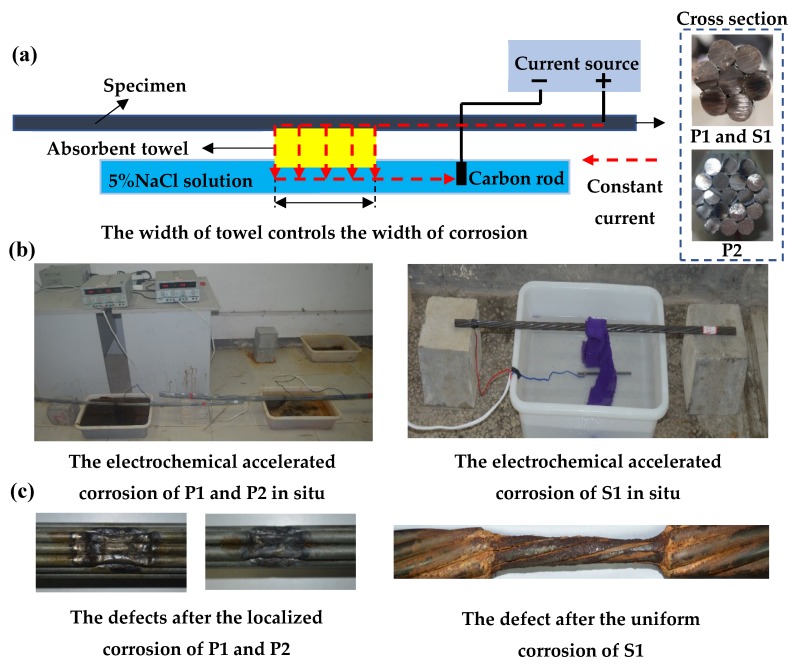
Electrochemical accelerated corrosion method: (**a**) Schematic diagram of corrosion circuit layout and the cross-section of the actual specimens; (**b**) the picture of corrosion in situ; (**c**) the defects after corrosion of the specimens. P1: the parallel wire strand made of seven steel wires; P2: the parallel wire strand made of 19 steel wires; S1: the steel strand.

The corrosion was carried out by the electrochemical accelerated corrosion system developed independently. The corrosion system is mainly composed of the constant-current device, 5%NaCl solution, water absorbent towel and carbon rod, as shown in Figure 15a. The positive pole of the current source is connected to the one end of the specimen, and the negative pole is connected to the carbon rod and placed in the NaCl solution. The current flows out from the positive pole through the current source, passing through the specimen, the towel, the NaCl solution, the carbon rod and return to the negative pole, which forms a loop. After being soaked in NaCl solution in advance, the current of the absorbent towel can produce a large amount of heat at the contact surface of the towel and the specimen through electrochemical corrosion reaction. At the same time, with the consumption of water, it will be automatically replenished by the absorbent towel to form an automatic corrosion reaction. Therefore, as long as the current exists, electrochemical corrosion will not stop.

According to Faraday’s law of electrolysis, so it can be expressed as:

(7) M=kIt,

where the *M*, *k*, *I*, *t* is the mass lost by the metal due to corrosion, the proportionality constant, the current intensity and the electrification time, respectively. In the experiment, the control corrosion current was set as 0.5 A, and the width of corrosion was determined by the contact width between the absorbent towel and the specimen. The corrosion time was set according to Equation (7), as shown in Table 3. In order to study the detection of corrosion width in this paper, it is necessary to control the consistent corrosion depth of P1 and P2 in each corrosion stage in the experiment. Due to the limitation of cross sections of P1 and P2, when binding the absorbent towel, there are only two wires at the contact between P1 and the absorbent towel, while three wires at the contact between P2 and the absorbent towel. Therefore, only when the power on time is also 2:3 can the corrosion depth of P1 and P2 be consistent. The system of corrosion in situ and corrosion marks is shown in Figure 15b. The defects after corrosion of the specimens are shown in Figure 15c. Among them, P1 and P2 adopted localized corrosion, and S1 adopted uniform corrosion, which the corroded sections observed from a two-dimensional angle were close to the rectangular section. 

**Figure 16:**
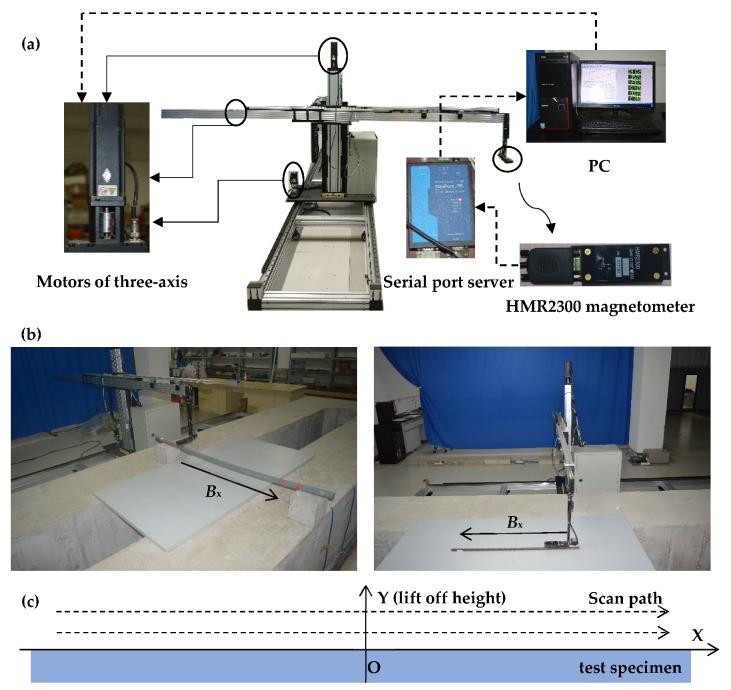
Magnetic signal acquisition system and acquisition path: (**a**) the composition of magnetic signal acquisition system; (**b**) the picture of magnetic signal acquisition of specimens in situ; (**c**) the Schematic diagram of magnetic signal acquisition path. *B*_x_: the magnetic flux component of *x* direction.

The collection of magnetic signals is mainly completed by the three-dimensional magnetic signal scanning device. The scanning system is mainly composed of three axis transmission device, magnetic signal sensor, serial port server and computer, as shown in Figure 16a. The transmission device is driven by three motors and can provide three mutually perpendicular scanning paths and record the current position of the magnetic sensor. The device’s magnetic signal sensor is the Honeywell HMR2300 three-dimensional magnetic flux leakage signal collector, which is a giant magneto-resistance sensor with a range of ±2 gauss and a resolution of about 70 micro-gauss. The length, width and height of the sensor is 8.2 cm, 3.6 cm and 2.5 cm respectively. Because the relation between flux density and magnetic field intensity is satisfied:

(8) $$H_{x}=\mu_{0}B_{x},$$

where the $\mu_{0}$ (the permeability of vacuum) is the constant, so the change rules of the *B*_x_ (flux density) and *H*_x_ are consistent. Therefore, the *B*_x_ can be used as SMFL magnetic signal for analysis in the experiment. Finally, the magnetic flux density data obtained is transmitted back to the PC through the serial port server then received and processed by the software developed by our team. The actual scanning situation of the sample is shown in Figure 16b. The direction along the length of the specimen was considered as the *X*-axis, which the magnetic signal component in this direction was considered as *B*_x_. The scanning path was set along the *x* direction and repeated along different *y*. The scanning length of P1, P2 and S1 was set as 80 cm, 80 cm and 40 cm. The *y* of P1, P2 and S1 was set as 1 cm to 6 cm respectively, which the spacing was set as 1 cm, as shown in Figure 16c.

### 3.2. Magnetic Signal Acquisition Results and Discussion

**Figure 17:**
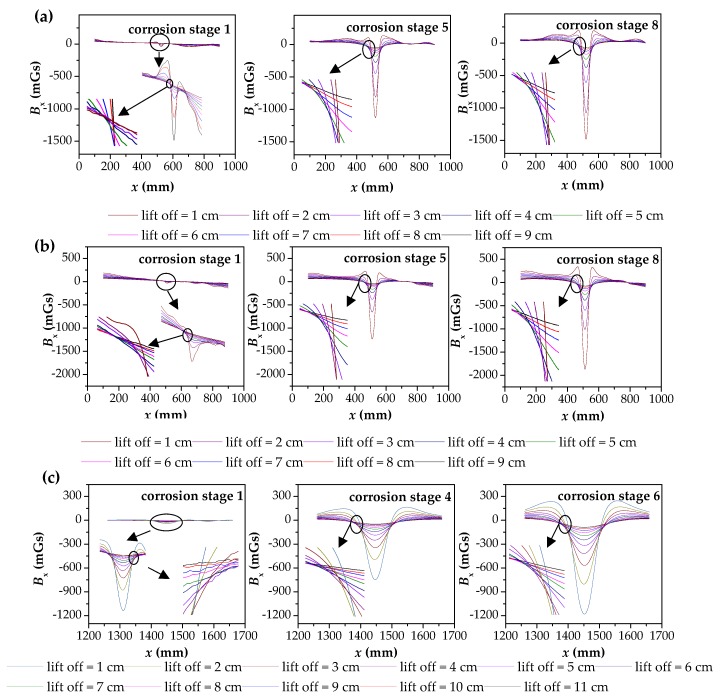
The obtained results of magnetic flux signals of three specimens at different corrosion stages: (**a**) the experimental results of P1 in corrosion stage 1, 5 and 8; (**b**) the experimental results of P2 in corrosion stage 1, 5 and 8; (**c**) the partial experimental results of S1 in corrosion stage 1, 4 and 6. P1: the parallel wire strand made of seven steel wires; P2: the parallel wire strand made of 19 steel wires; S1: the steel strand; *B*_x_: the magnetic flux component of *x* direction; *x*: the coordinate along the length of the specimens.

**Figure A1:**
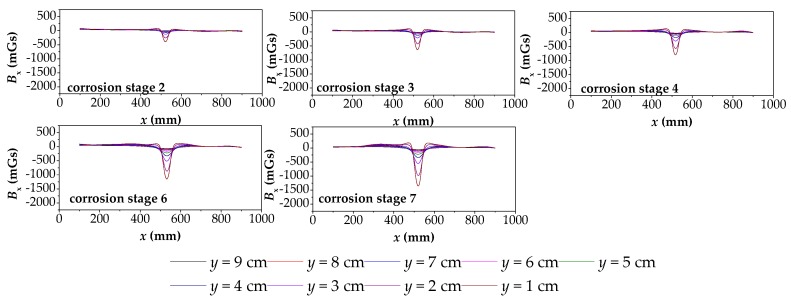
The *B*_x_ curves of P1 under residual corrosion stages. P1: the parallel wire strand made of seven steel wires; *B*_x_: the magnetic flux component of *x* direction; *x*: the coordinate along the length of the specimens.

**Figure A2:**
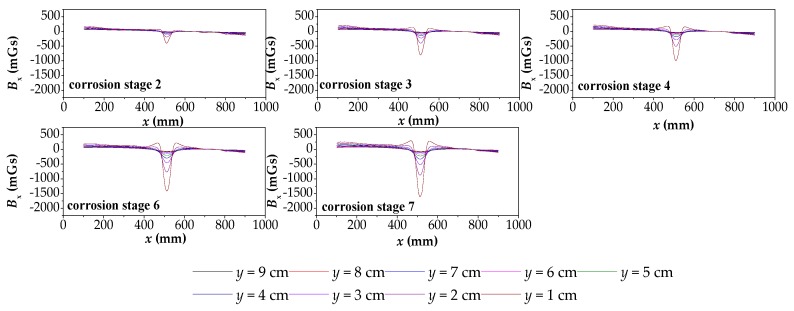
The *B*_x_ curves of P2 under residual corrosion stages. P2: the parallel wire strand made of 19 steel wires; *B*_x_: the magnetic flux component of *x* direction; *x*: the coordinate along the length of the specimens.

**Figure A3:**
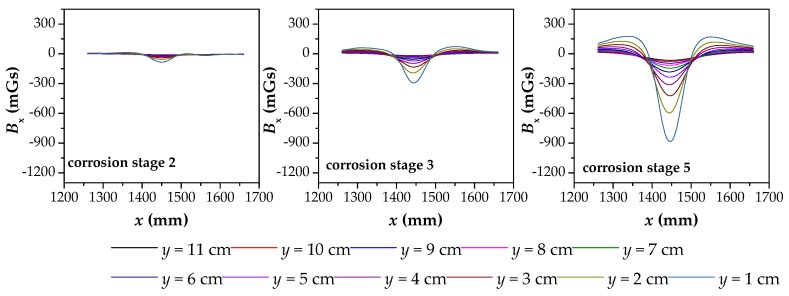
The *B*_x_ curves of S1 under residual corrosion stages. S1: the steel strand; *B*_x_: the magnetic flux component of *x* direction; *x*: the coordinate along the length of the specimens.

SMFL magnetic signal data of samples P1, P2 and S1 under all corrosion conditions were collected, and the signal components *B*_x_ along the length of the specimen were extracted. Now three corrosion stages selected for P1, P2 and S1 were described respectively. The relation between the *B*_x_ and the coordinate *x* is shown in Figure 17a–c. The other corrosion stages of relation between the *B*_x_ and the coordinate *x* of P1, P2 and S1 are shown in Figure A1, Figure A2 and Figure A3, respectively.

In Figure 17, multiple images are analyzed. All curves show a trend of *B*_x_ first decreasing and then increasing with the increase of *x*, which is the SMFL phenomenon at the defect. At the corrosion stage 1, the *B*_x_ curves in the image are almost a straight line near 0. After magnified, the *B*_x_ curves at the corrosion center (*x* = 500 mm for P1 and P2, *x* = 1450 mm for S1) has different degrees of the downward protrusion. With the increase of corrosion degree, the downward protuberance becomes more and more obvious, and the extreme point becomes larger and larger. Analysis of a single image, with the increase of the *y*, the law of *B*_x_ curves will not change. However, with the increase of the *y*, the phenomenon of protrusion of *B*_x_ curves becomes weak, and the extreme point becomes smaller. After analysis, it is found that the phenomena presented by the above experiments are consistent with the laws obtained from the theoretical model.

In order to more clearly observe the intersection of each curve, the position of the intersection of each curve in each image is enlarged. It can be seen that after a certain degree of corrosion, the intersection of multiple curves is consistent with the situation shown in Figure 2. For the P1, P2 specimens, in the first corrosion stage, a galvanized layer is applied to the surface of the parallel wire as a protective layer, which caused the actual corrosion degree to be far less than the corrosion degree of theory. Hence, the phenomenon of extreme value reflected in the image is not obvious, which leads to the existence of multiple disjoint curves, and it will not be considered in the subsequent analysis. However, as the degree of corrosion increases, the phenomenon that the intersection point does not exist will disappear, and the same phenomenon also exists for the first two corrosion stages of S1, as shown in the enlarged image of corrosion stage 1 in Figure 17.

**Figure 18:**
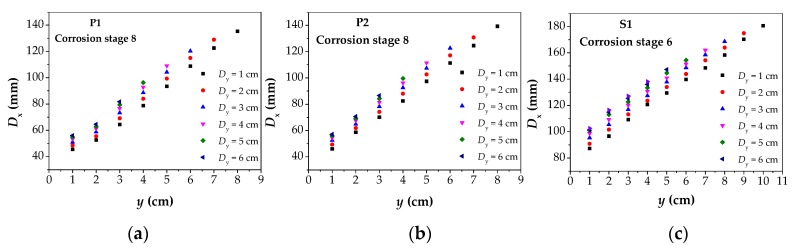
The obtained values of *D*_x_ of the three specimens in a corrosion stage: (**a**) the values of *D*_x_ of P1 in corrosion stage 8; (**b**) the values of *D*_x_ of P2 in corrosion stage 8; (**c**) the values of *D*_x_ of S1 in corrosion stage 6. P1: the parallel wire strand made of seven steel wires; P2: the parallel wire strand made of 19 steel wires; S1: the steel strand; *D*_x_: the intersecting point distance between curves of magnetic flux component of *x* direction at different lift off heights.

Then, further analysis was made to find out the *D*_x_ values under different corrosion conditions through the method flow in Figure 10, and the *D*_x_–*y* diagram is drawn, as shown in Figure 18. Because all data were processed in the same way, only the last corrosion stage of P1, P2 and S1 specimens were selected for detailed descriptions. As can be seen from Figure 18, with the increase of *y*, the values of *D*_x_ show a trend of linear increase with a high degree of linearity. Under all values of *D*_y_, the rule between *D*_x_ and *y* is the same, which is the same as the result obtained in the theoretical analysis (Figure 9), further illustrating the correctness of the theoretical model and the prediction of corrosion width by this method. Moreover, by observing each image separately, it can be known that when *y* is very small, the values of *D*_x_ are very close to the actual corrosion width (the corrosion width of P1 and P2 is 40 mm; the corrosion width of S1 is 80 mm).

**Figure 19:**
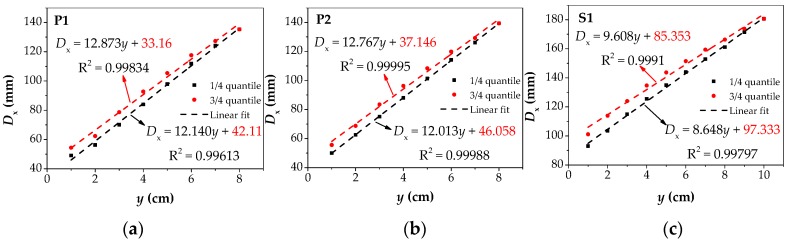
The Linear fitting results of *D*_x_ of the three specimens: (**a**) the fitting results of P1; (**b**) the fitting results of P2; (**c**) the fitting results of S1. P1: the parallel wire strand made of seven steel wires; P2: the parallel wire strand made of 19 steel wires; S1: the steel strand; the red text in the graph represents the predicted value of corrosion width. *D*_x_: the intersecting point distance between curves of magnetic flux component of *x* direction at different lift off heights.

After integrating all the data under the value of *D*_y_, the 1/4 and 3/4 quantiles of the data were fitted linearly. The fitting results are shown in Figure 19. The black points and dotted lines respectively represent the value of the 1/4 point and the corresponding linear fitting curve, while the red points represent the value of the 3/4 point and the corresponding linear fitting curve. It can be found that the three samples all follow a relatively good linear distribution when fitting, and the values of R^2^ all exceed 0.998. The equation of the corresponding relation between *D*_x_ and *y* can be expressed by the fitted curve, and its intercept represents the value of *D*_x_ at *y* = 0, which is the predicted value of the corrosion width (marked in red font). There are two predicted values in each figure, and the upper and lower limits of the predicted values are used to represent the possible ranges of the true values.

**Figure 20:**
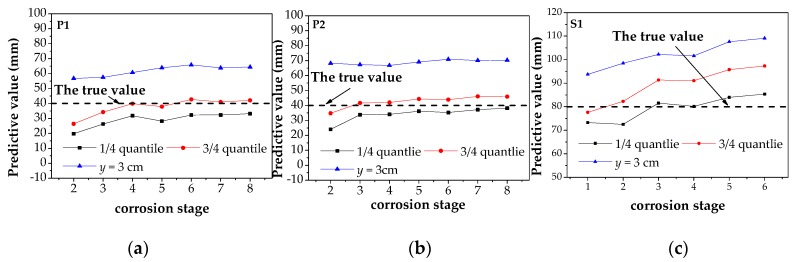
The predicted results of corrosion width of three specimens under different corrosion stages: (**a**) the predicted results of P1; (**b**) the predicted results of P2; (**c**) the predicted results of S1. P1: the parallel wire strand made of seven steel wires; P2: the parallel wire strand made of 19 steel wires; S1: the steel strand.

The predicted corrosion width in all corrosion stages obtained by fitting was compared with *y* = 3 cm and the true values, as shown in Table A1. As can be seen from Table A1, most of the results are much larger than the true corrosion width when *y* = 3 cm, while most of the errors between the result obtained by this method and the real value are within 20%. However, for the three specimens in the small corrosion degree, the prediction result is lower than the true values, which can be explained by the rule that is shown in Figure 10. Meanwhile, the experimental results are consistent with the theoretical analysis results. In order to more intuitively represent the errors of prediction results, the calculation results in Table A1 are drawn into graphs for analysis, as shown in Figure 20.

In Figure 20, the vertical coordinate represents the predictive values of corrosion width, and the horizontal coordinate represents the corrosion stage. Meanwhile, the fitting results of the 1/4 quantile and the 3/4 quantile, the values of *D*_x_ when *y* = 3 cm and the true corrosion width are represented by the black line, the red line, the blue line and the dotted line respectively. It can be seen from the figure that the predicted value of corrosion width increases slowly with the increase of corrosion degree, which is consistent with the *D*_x_–*h* image in the theoretical model. At the same time, it can be found that the predicted values obtained by fitting are basically distributed near the true values, and the accuracy is significantly improved compared with the measured value of *y* = 3 cm. The experimental results show that this method can improve the accuracy of corrosion detection.

## 4. Conclusions

In order to quantitatively study the detection method of corrosion width of cables, the corrosion detection experiment was carried out by numerical analysis of the theoretical model and taking parallel steel wire and steel strand, commonly used materials of cables, as the research object. The following conclusions can be drawn:

1. A two-dimensional magnetic dipole model was established, which considered the continuous distribution of bottom magnetic charge density. By numerical analysis with MATLAB, it was found that the intersection points of *H*_x_ curves at different lift off heights were distributed near the boundary of the corrosion region. Then the parameter *D*_x_ was put forward as an index to predict the corrosion width.
2. Further analysis on the influencing factors of the parameters *b*, *h*, *y* and *D*_y_ on *D*_x_ is carried out. It shows that under different *b* and *h*, the *D*_x_ has obvious correspondence with the corrosion width, and the law is stable, which indicates that the *D*_x_ can be used as an index to judge the corrosion width. The *D*_x_–*y* curves exist an obvious linear relationship, and the *D*_x_ can basically converge to the true corrosion width when *y* = 0. Hence, the corrosion width can be quantitatively detected by linear fitting of the *D*_x_–*y* curves.
3. The 1/4 and 3/4 quantiles of *D*_x_–*y* curves under different values of *D*_y_ are used for linear fitting. The fitted intercept is used as the detection method for the predicted value of the range of corrosion width. The corrosion detection tests of parallel steel wire bundles and steel strand are carried out. The experimental results verified that this method can effectively improve the detection accuracy of corrosion width of concealed engineering (such as the existence of cable sheath which limits the corrosion detection of cables with minimum lifting height).
4. Based on the theory of SMFL, a new method of the detection of corrosion width has been proposed. Compared with conventional corrosion detection methods, this technology has the advantages of simple equipment, lower cost and more accurate quantification of corrosion width. It improves the existing methods of detection of corrosion size, which has certain research value.

## Figures and Tables

**Table 1:** Prediction results and error table.

Fitting Situation	Ture Value	Measurements of *D*_x_ When *y* = 3 cm	Measurements Error of *D*_x_ When *y* = 3 cm	Predictive Value	The Prediction Error
1/4 quantile	2 cm	5.42	171%	1.58	−21%
3/4 quantile	2 cm	5.42	171%	2.80	40%

**Table 2:** The parameters of specimens.

Specimens	Number	Corrosion Length	Corrosion Method
Parallel wire strand	P1	4 cm	Electrochemical localized corrosion
	P2		
Steel strand	S1	8 cm	Electrochemical uniform corrosion

**Table 3:** The corrosion schedule of all specimens (unit: h).

	Stage	0	1	2	3	4	5	6	7	8
Number										
P1		0	4	8	12	16	20	24	28	32
P2		0	6	12	18	24	30	36	42	48
S1		0	12	24	36	48	60	72	-	-

## Appendix A

**Table A1:** The calculation sheet of error.

Number	Corrosion Stage	True Value/mm	*D*_x_ When *y* = 3 cm		1/4 Quantile Fit		3/4 Quantile Fit	
			Results/mm	Error	Results/mm	Error	Results/mm	Error
P1	1	40	-	-	-	-	-	-
	2		56.8	0.42	19.78	0.5055	26.4	0.34
	3		57.5	0.4375	26.28	0.343	34.26	0.1435
	4		60.7	0.5175	31.79	0.2053	39.91	0.0023
	5		63.9	0.5975	28.25	0.2938	37.91	0.0523
	6		65.8	0.645	32.3	0.1925	42.74	0.0685
	7		63.8	0.595	32.34	0.1915	40.99	0.0248
	8		64.4	0.61	33.16	0.171	42.11	0.0528
P2	1	40	-	-	-	-	-	-
	2		68.2	0.705	24.02	0.3995	34.8	0.13
	3		67.3	0.6825	33.74	0.1516	41.65	0.0413
	4		66.8	0.67	34.06	0.1485	42.01	0.0503
	5		69.1	0.7275	36.15	0.0963	44.33	0.1083
	6		70.9	0.7725	35.29	0.1178	43.9	0.0975
	7		70.1	0.7525	37.15	0.0713	46.06	0.1515
	8		70.3	0.7575	38.19	0.0453	45.92	0.148
S1	1	80	93.7	0.1713	73.25	0.0844	77.62	0.0298
	2		98.5	0.2313	72.52	0.0935	82.27	0.0284
	3		102.2	0.2775	81.52	0.019	91.34	0.1418
	4		101.6	0.27	80.14	0.0018	91.07	0.1384
	5		107.6	0.345	84	0.05	95.76	0.197
	6		109.1	0.3638	85.35	0.0669	97.33	0.2166

## Appendix B

**Table A2:** The parameter list of MATLAB finite element calculation in Figure 2.

*b*/cm	*h*/cm	$\rho_{\max}/\mu_{0}$	*x*/cm	*y*/cm
4	2	1	−10 to 10 Step = 0.1	*y* = 1
				*y* = 2
				*y* = 3
				*y* = 4
				*y* = 5
				*y* = 6
				*y* = 7
				*y* = 8

**Table A3:** The parameter list of MATLAB finite element calculation in Figure 4.

*b*/cm	*h*/cm	$\rho_{\max}/\mu_{0}$	*x*/cm	*y*_1_/cm	*y*_2_/cm
1	2	1	−10 to 10 Step = 0.1	1 to 99 Step = 1	2 to 100 Step = 1

The *y*_1_ and *y*_2_ are arbitrarily combined within the range of values, but *y*_1_ is not equal to *y*_2_.

**Table A4:** The parameter list of MATLAB finite element calculation in Figure 5.

*b*/cm	*h*/cm	$\rho_{\max}/\mu_{0}$	*x*/cm	*y*_1_/cm	*y*_2_/cm
1	2	1	−10 to 10 Step = 0.1	1 to 99 Step = 1	2 to 100 Step = 1
2					
3					
4					
5					
6					

The *b*, *y*_1_, and *y*_2_ are arbitrarily combined within the range of values, but *y*_1_ is not equal to *y*_2_.

**Table A5:** The parameter list of MATLAB finite element calculation in Figure 8.

*b*/cm	*h*/cm	$\rho_{\max}/\mu_{0}$	*x*/cm	*y*_1_/cm	*y*_2_/cm
4	1	1	−10 to 10 Step = 0.1	1 to 99 Step = 1	2 to 100 Step = 1
	2				
	3				
	4				
	5				
	6				

The *h*, *y*_1_, and *y*_2_ are arbitrarily combined within the range of values, but *y*_1_ is not equal to *y*_2_.
